# Supplementary material for: Encoding of multi-modal emotional information via personalized skin-integrated wireless facial interface
Source: Nat Commun. 2024 Jan 15;15:530. doi: 10.1038/s41467-023-44673-2 (PMC10789773; doi:10.1038/s41467-023-44673-2)
Supplement: Supplementary file 1 — Supplementary Information [file 41467_2023_44673_MOESM1_ESM.pdf]

# Supplementary materials

## Encoding of multi-modal emotional information via personalized skin-integrated wireless facial interface

*Jin Pyo Lee[1,2†], Hanhyeok Jang[1], Yeonwoo Jang[1], Hyeonseo Song[1], Suwoo Lee[1], Pooi See Lee[2]*, and Jiyun Kim[1,3]**

*[1] School of Material Science and Engineering, Ulsan National Institute of Science and Technology, Ulsan 44919, South Korea*
*[2] School of Materials Science and Engineering, Nanyang Technological University, Singapore 639798, Singapore*
*[3] Center for Multidimensional Programmable Matter, Ulsan National Institute of Science and Technology, Ulsan 44919, South Korea*
*Corresponding author. Email: pslee@ntu.edu.sg (P.S.L.); jiyunkim@unist.ac.kr (J.K.)

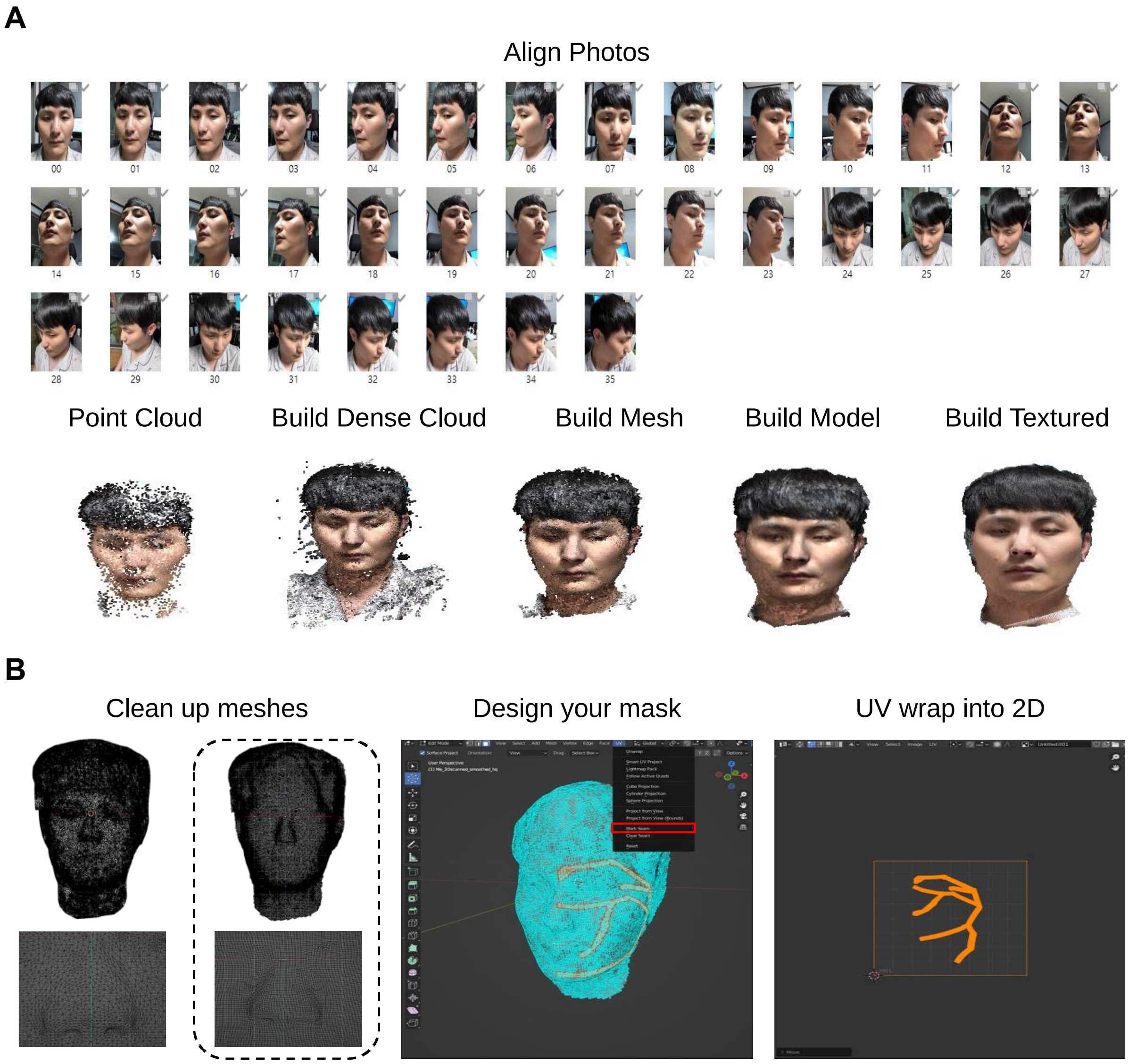


**Supplementary fig. 1 | Fabrication process of personally customized wearable mask based on 3D scanning and computer design tools a,** Schematics of 3D scanning method which enables us to acquire the 3d model by utilizing photos taken from multiple angles. **b,** Post-processing steps for 2d drawing implemented by computer design tools.

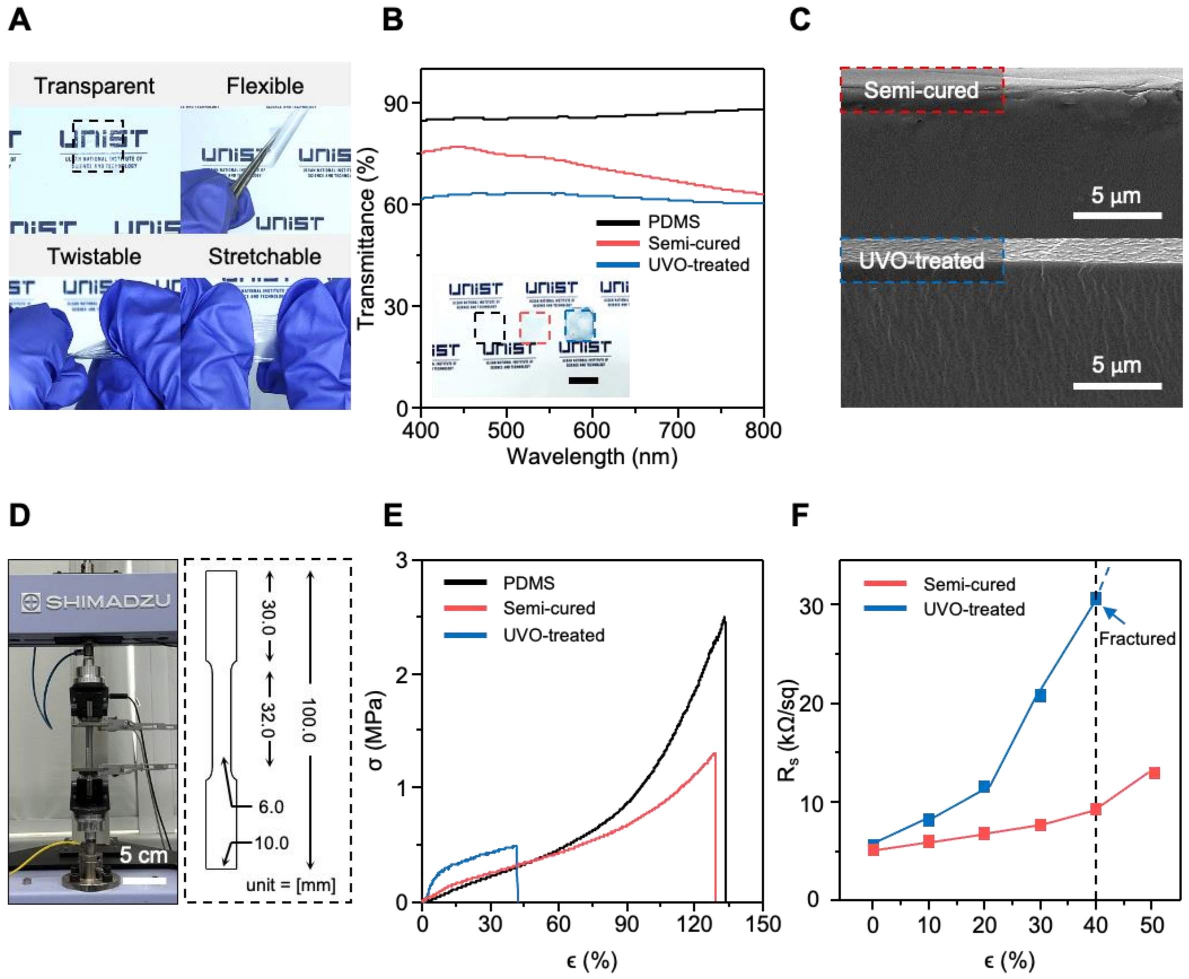


**Supplementary fig. 2 | Characterization and performance of the PEDOT:PSS embedded** **a,** Photographs of the as-prepared electrode showing transparent, flexible, twistable and stretchable characteristics. **b**, Transmittance spectra of the pure PDMS film, the PEDOT:PSS/UVO-treated PDMS film and PEDOT:PSS/semi-cured PDMS film in the visible wavelength range from 400 to 800 nm. **c**, SEM images of a PEDOT:PSS/semi-cured PDMS film (top) and PEDOT:PSS/UVO-treated PDMS film (bottom). Scale bar: 5 μm **d,** Experimental set-up of tensile testing (right) and specimen geometry of the tested conductor (left). **e**, Stress-strain behavior of free-standing PEDOT:PSS/UVO-treated PDMS film and PEDOT:PSS/semi-cured PDMS film. **f**, Sheet resistances of the PEDOT:PSS/semi-cured PDMS and PEDOT:PSS/UVO-treated PDMS film under various tensile strains. Film thickness are around 100 μm.

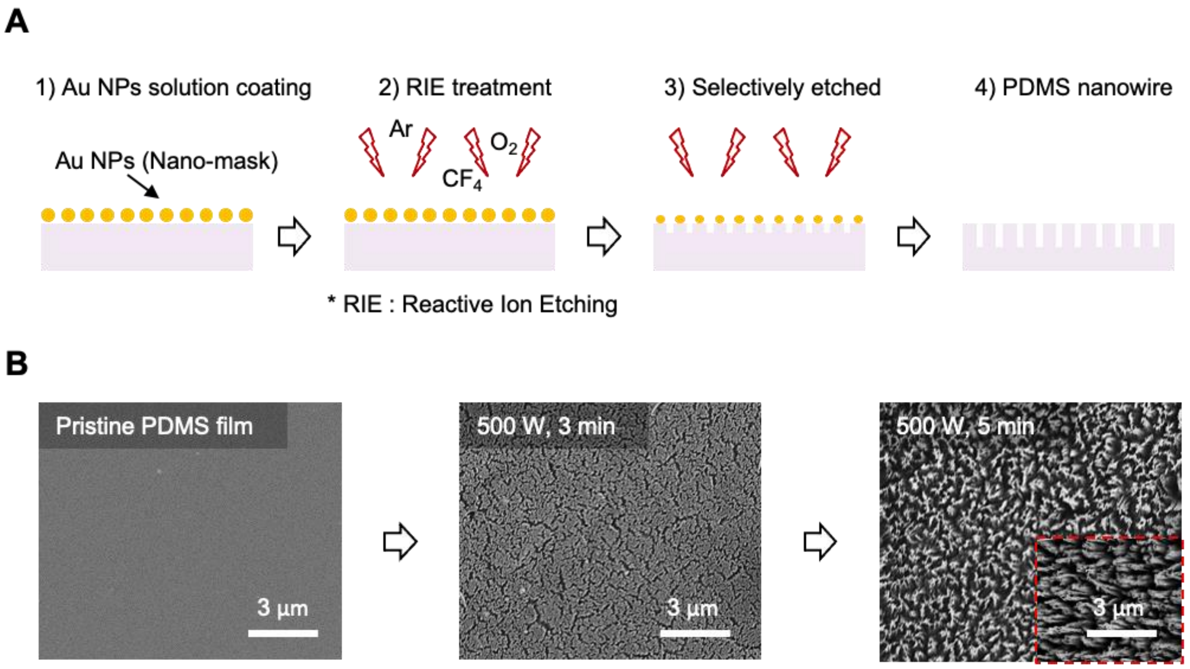


**Supplementary fig. 3 | Fabrication process of nanostructure formation on the dielectric surface a,** Schematic images of fabrication process for the aligned dielectric nanowires created by one-step inductively coupled plasma (ICP) process. **b,** SEM images showing the polymer NWs formation depend on the processing time.

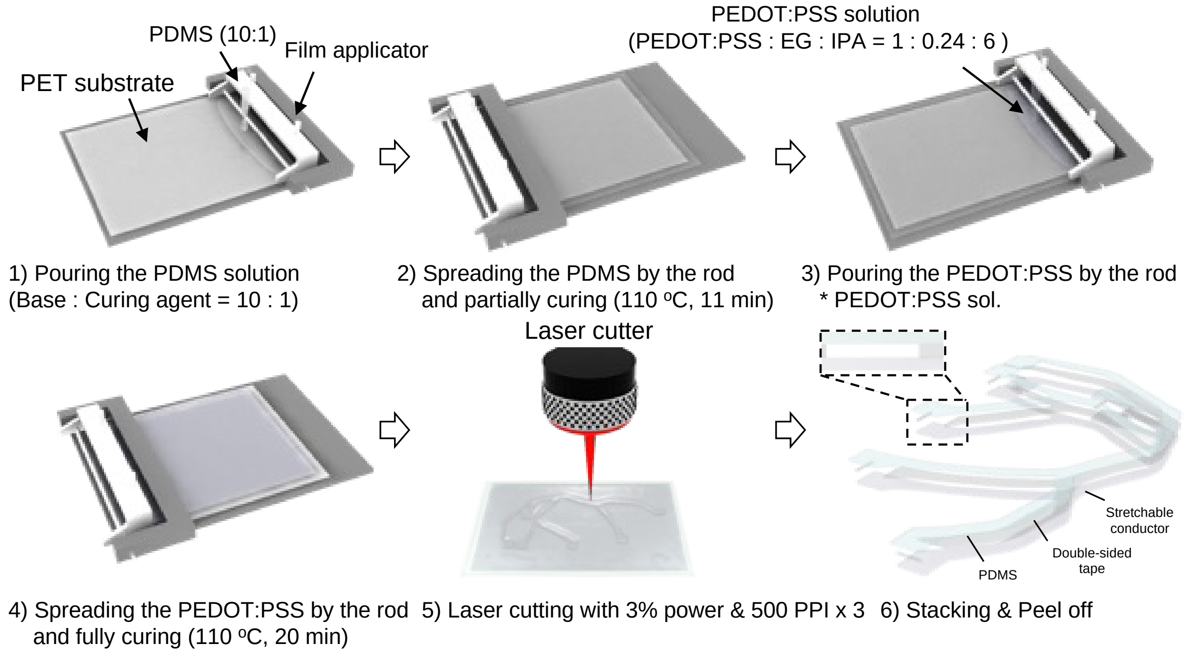


**Supplementary fig. 4 | Schematic diagram of fabrication process for the stretchable electrode and wearable mask**

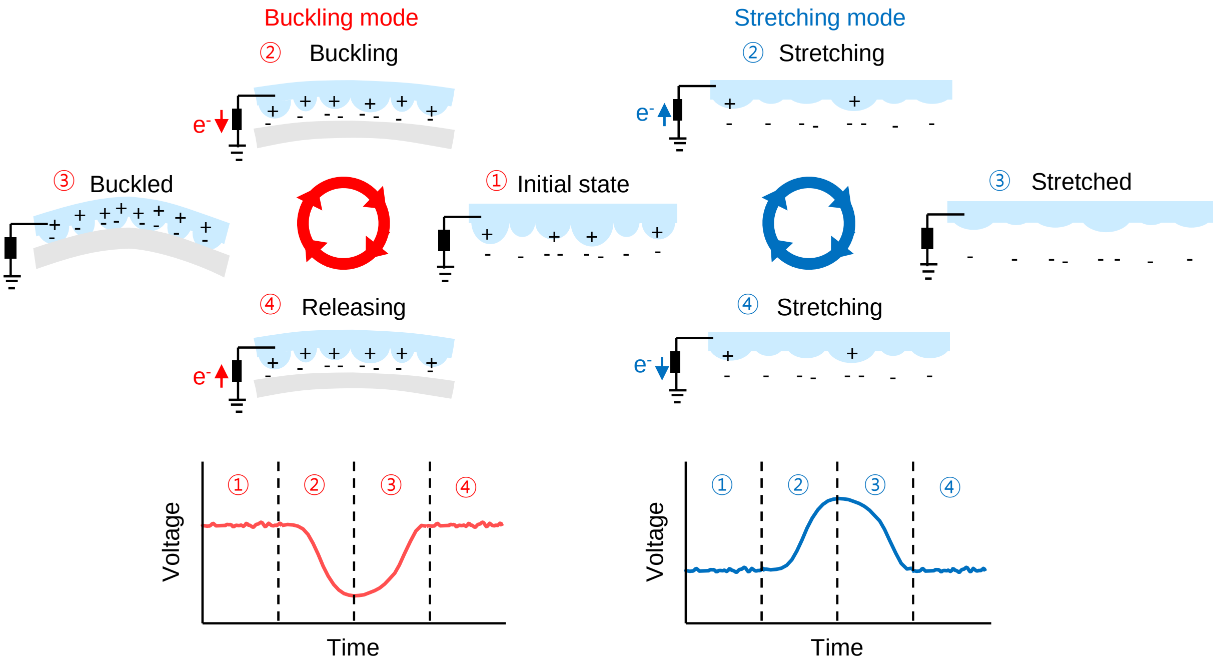


**Supplementary fig. 5 | Detailed working mechanism for the generation of output voltage and current in our bidirectional triboelectric strain sensor during the buckling (left) and stretching cycle (right)**

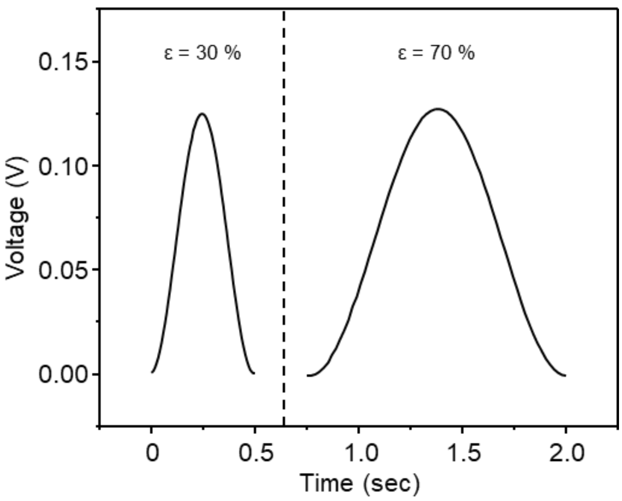


**Supplementary fig. 6 | Comparison of the output voltage with 30% strain and 70% strain under working frequency of 0.5 Hz.**

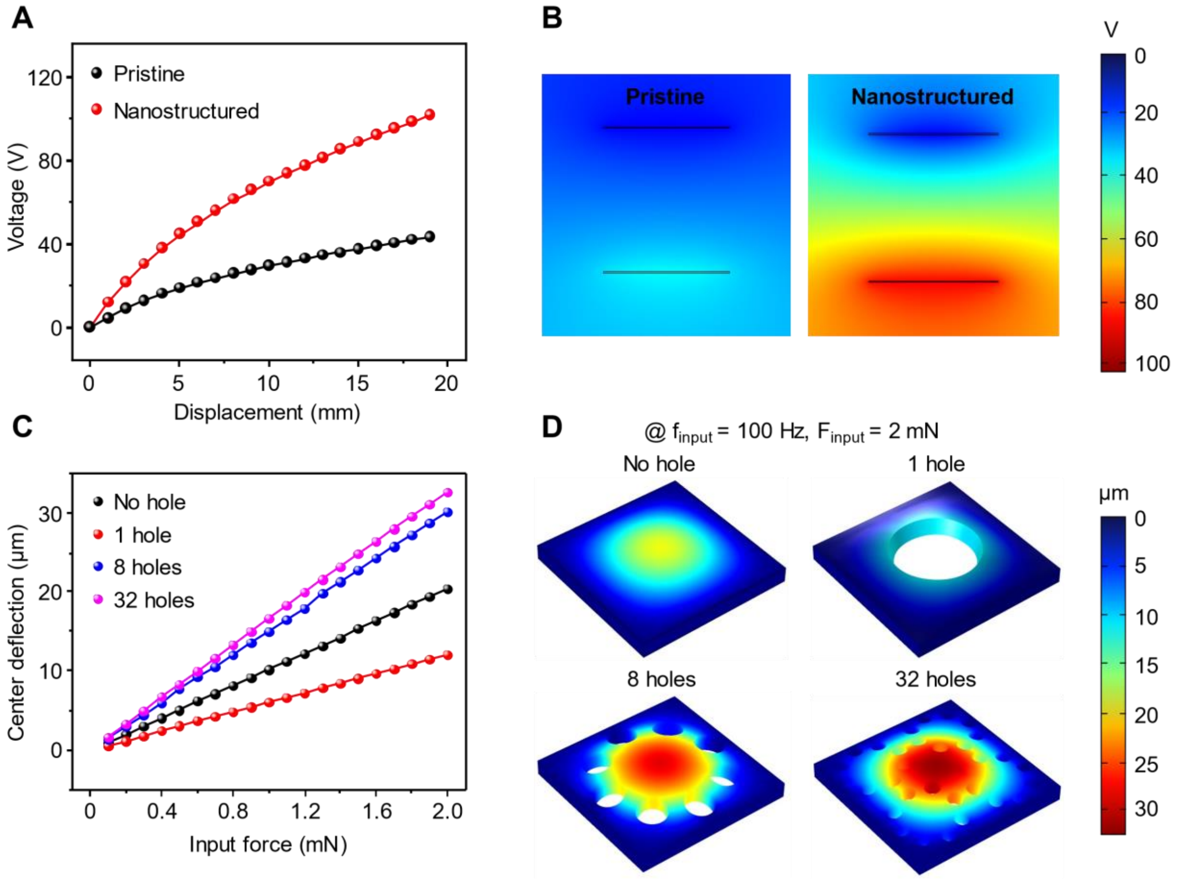


**Supplementary fig. 7 | Simulation modelling and analysis using COMSOL software a,** Calculated $V_{oc}$ depending on the gap distance. **b,** Calculated electrical potential distribution at maximum separated position. **c,** Calculated deflection at the center of the diaphragm with various number of holes. **d,** Deflection distribution of the diaphragm with various number of holes calculated under input frequency of 100 Hz and input force of 2 mN.

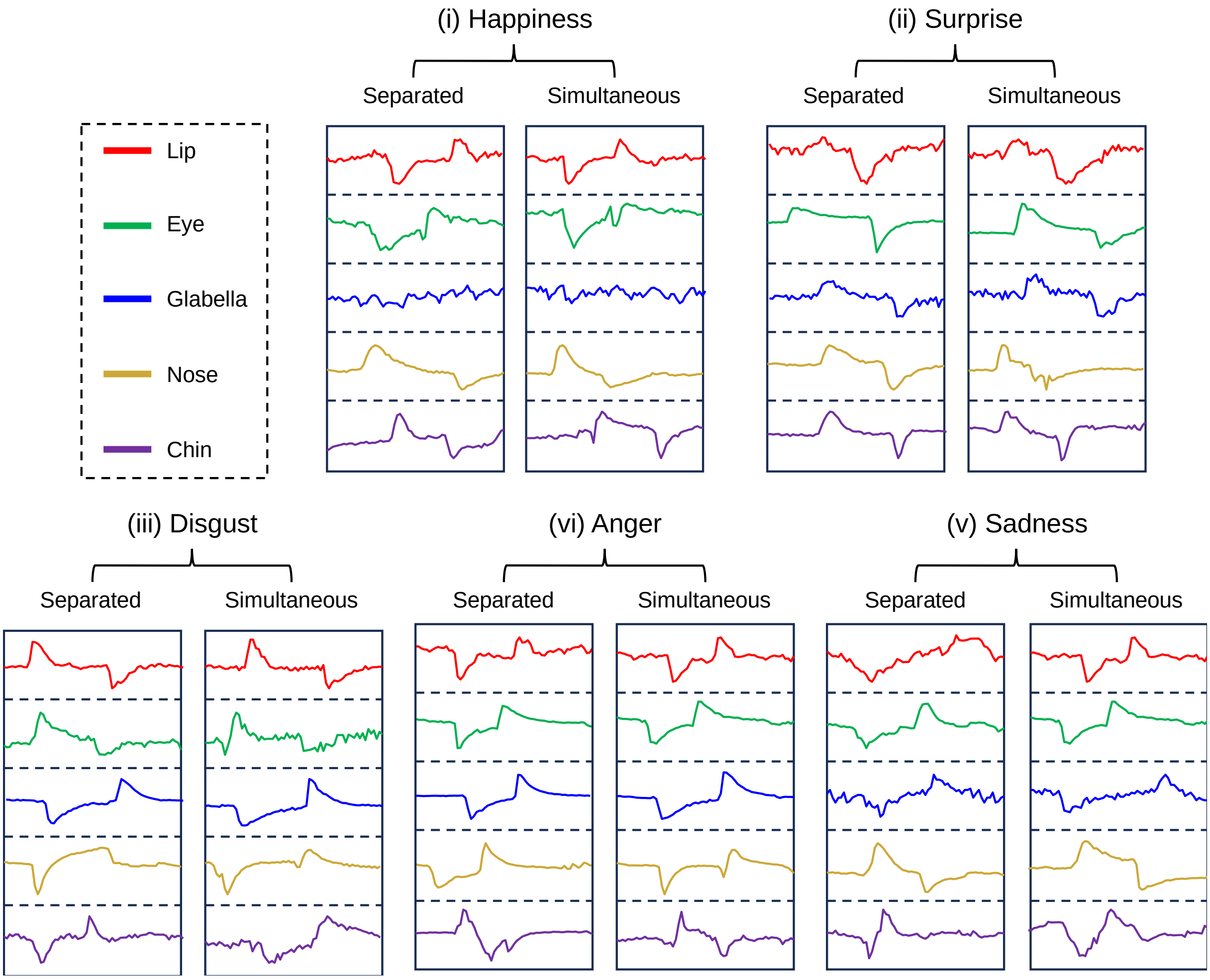


**Supplementary fig. 8 | Comparison of the output voltage signals from multi-channel sensors under various facial expressions (i-happiness, ii-surprise, iii-disgust, vi-anger, v-sadness) when they were measured separately or simultaneously with vocal expressions.**

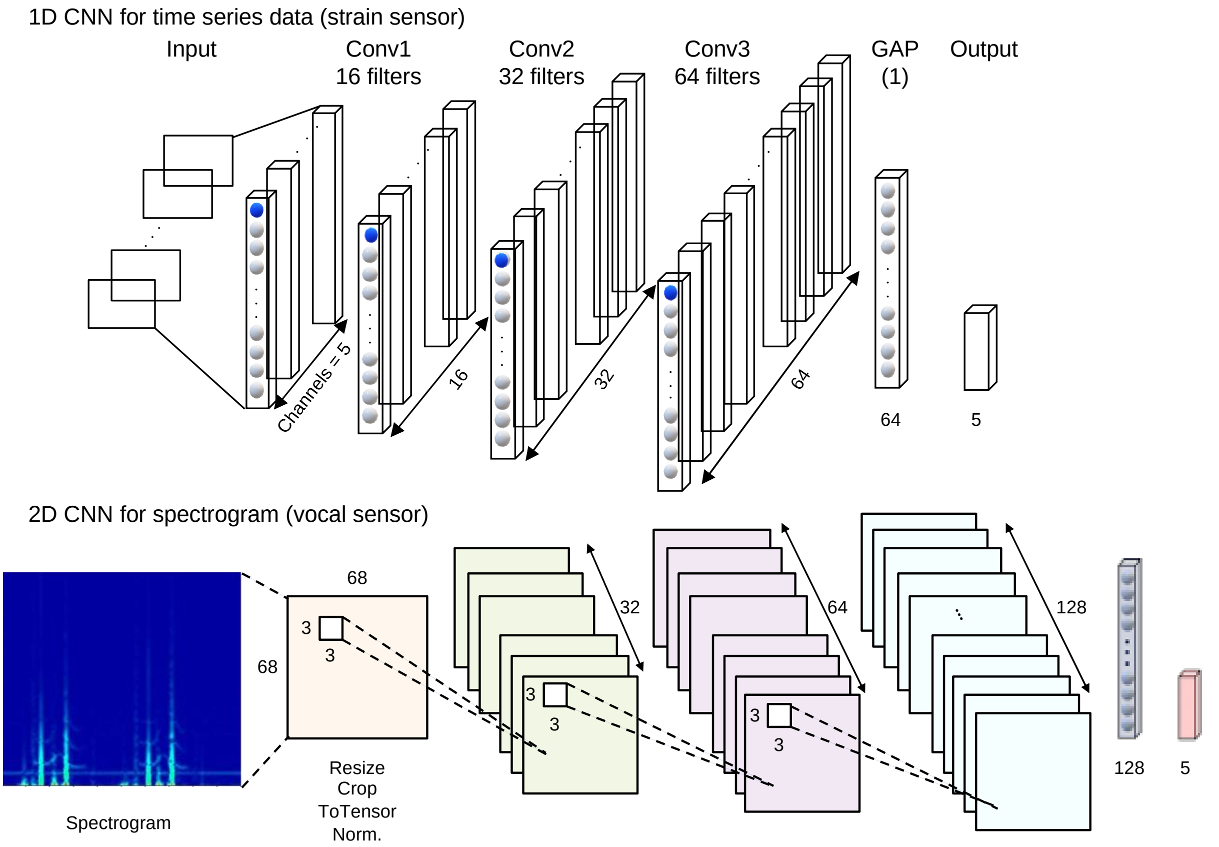


**Supplementary fig. 9 | Schematic illustration of our CNN based model used for classification of verbal/non-verbal expression.**

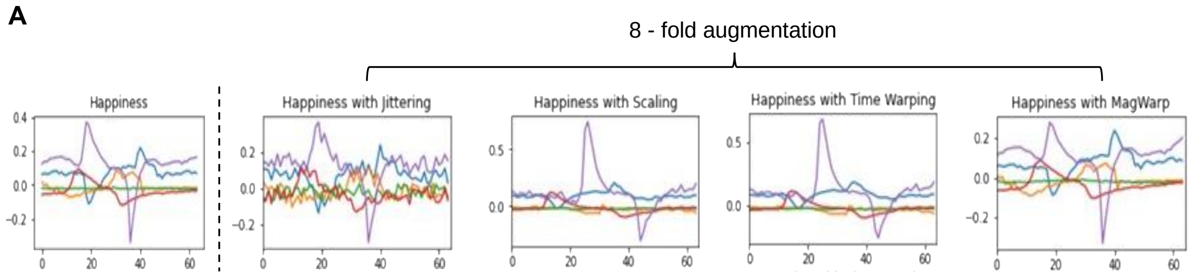


| | CNN | Jittering | Scaling | Time warping | Mag. warping |
|---|---|---|---|---|---|
| Train | 100.00 | 100.00 | 100.00 | 100.00 | 100.00 |
| Test | 86.67 | 90.00 | 93.33 | 86.67 | 90.00 |

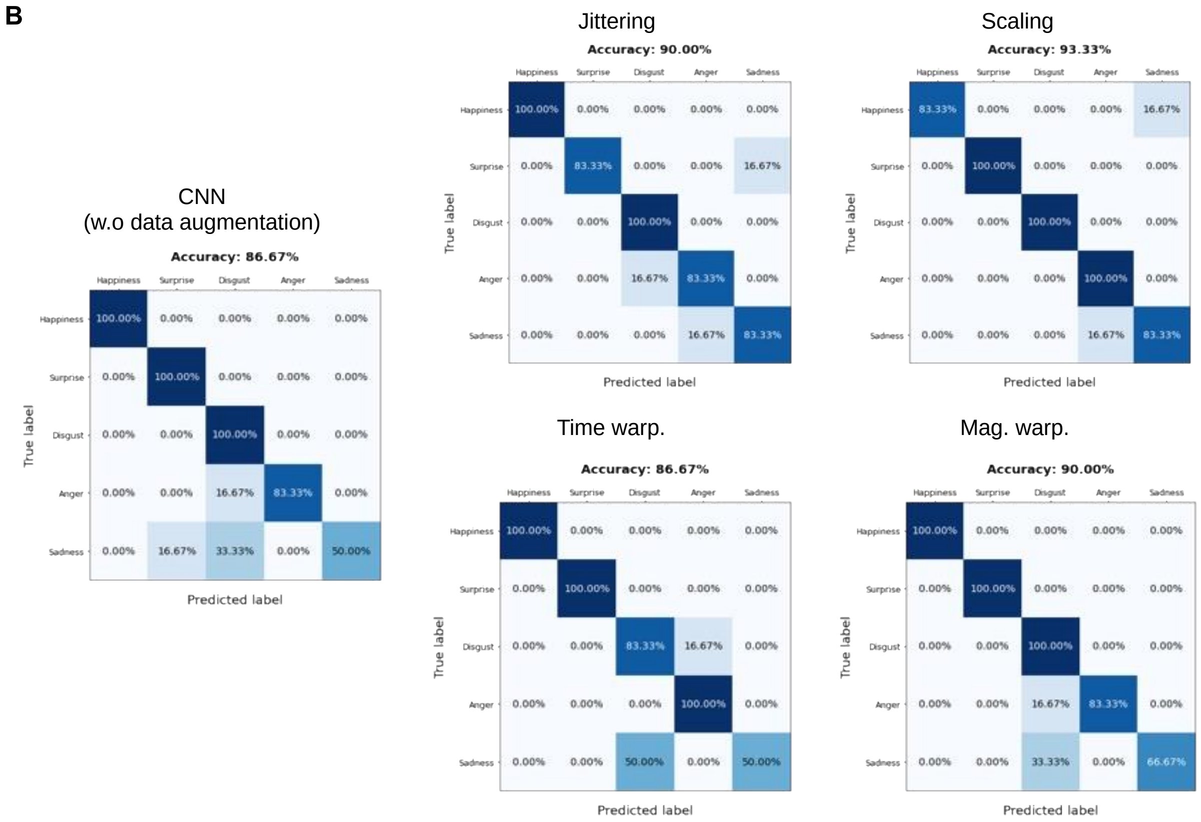


**Supplementary fig. 10 | Application of data augmentation technique for enhancement in training a,** various data augmentations that used in our experiment: jittering, scaling, time warping, magnitude-warping methods. **b,** The comparison on confusion matrixes among the corresponding data augmentation techniques.

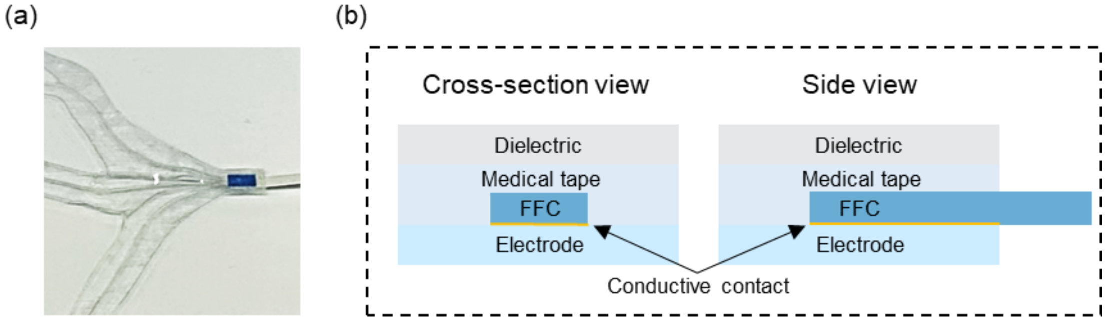


**Supplementary fig. 11 | Detailed information on electrical connection for data acquisition.** **a,** enlarged view of the photograph for wiring and **b** schematic images for the electrical connection in cross-section and side view, respectively.

**Table S1 | Parameters utilized in the theoretical calculation of the sensors.**

| Common parameters | | | | |
|---|---|---|---|---|
| PEDOT:PSS | Length (mm) | | 10 | |
| | Width (mm) | | 10 | |
| | Thickness (mm) | | 0.1 | |
| | $\varepsilon_{PEDOT:PSS}$ | | 1000 | |
| Polydimethylsiloxane (PDMS) | Length (mm) | | 10 | |
| | Width (mm) | | 10 | |
| | Thickness (mm) | | 0.2 | |
| | $\varepsilon_{PDMS}$ | | 2.75 | |
| PDMS Nanowires (NWs) | Diameter (μm) | | 0.01 | |
| | Thickness (μm) | | 1 | |
| **Parameters for strain sensor** | | | | |
| Triboelectric surface charge density, σ (nC $m^{-2}$) | | | 50 | |
| Maximum separation distance, $x_{max}$ (mm) | | | 2 | |
| **Parameters for vibration sensor** | | | | |
| Hole diameters (mm) (Open ratio = 20%) | 1 hole | 8 holes | 32 holes | |
| | 2.52 | 0.89 | 0.45 | |

**Table S2 | The parameters for configuring Convolution Neural Network (CNN).**

| Layer | No. of Filters | Kernel size | Stride | Input Size | Output Size |
|---|---|---|---|---|---|
| Strain sensor (1D-CNN) | | | | | |
| Conv. Layer 1 | 16 | 3 | 1 | (None, 64, 5) | (None, 64, 16) |
| Batch Norm. | | | | (None, 64, 16) | (None, 64, 16) |
| Tanh | | | | | |
| Conv. Layer 2 | 32 | 3 | 1 | (None, 64, 32) | (None, 64, 32) |
| Batch Norm. | | | | (None, 64, 32) | (None, 64, 32) |
| Tanh | | | | | |
| Conv. Layer 3 | 64 | 3 | 1 | (None, 64, 64) | (None, 64, 64) |
| Batch Norm. | | | | (None, 64, 64) | (None, 64, 64) |
| GAP layer | | | | (None, 64, 64) | (None, 64) |
| F.C | | | | (None, 64) | (None, 5) |
| Softmax | | | | | |
| Vibration sensor (2D-CNN) | | | | | |
| Layer | No. of Filters | Kernel size | Stride | Input Size | Output Size |
| Conv. Layer 1 | 32 | 3 x 3 | 1 | (None, 68, 68, 3) | (None, 66, 66, 32) |
| Batch Norm. | | | | (None, 66, 66, 32) | (None, 66, 66, 32) |
| Tanh | | | | | |
| Conv. Layer 2 | 64 | 3 x 3 | 1 | (None, 66, 66, 32) | (None, 64, 64, 64) |
| Batch Norm. | | | | (None, 64, 64, 64) | (None, 64, 64, 64) |
| Tanh | | | | | |
| Conv. Layer 3 | 128 | 3 x 3 | 1 | (None, 64, 64, 64) | (None, 62, 62, 128) |
| Batch Norm. | | | | (None, 62, 62, 128) | (None, 62, 62, 128) |
| GAP layer | | | | (None, 62, 62, 128) | (None, 128) |
| F.C (128) | | | | (None, 128) | (None, 5) |
| Softmax | | | | | |

**Table S3 | Information of the dataset from PSIFI utilized in emotion recognition.**

| Pre-training | | | | | |
|---|---|---|---|---|---|
| Dataset | Train set | | Test set | Total | |
| | original | augmented | | original | augmented |
| Facial expression (non-verbal) | 70 | 560 | 30 | 100 | 590 |
| Vocal speech (verbal) | 70 | 560 | 30 | 100 | 590 |

| Transfer-learning | | | | | |
|---|---|---|---|---|---|
| Dataset | Train set | | Test set | Total | |
| | original | augmented | | original | augmented |
| Facial expression (non-verbal) | 35 | 270 | 15 | 50 | 285 |
| Vocal speech (verbal) | 35 | 270 | 15 | 50 | 285 |
