# Supplementary material for: Encoding of multi-modal emotional information via personalized skin-integrated wireless facial interface
Source: Nat Commun. 2024 Jan 15;15:530. doi: 10.1038/s41467-023-44673-2 (PMC10789773; doi:10.1038/s41467-023-44673-2)
Supplement: Supplementary file 3 — Description of Additional Supplementary Files [file 41467_2023_44673_MOESM3_ESM.pdf]

Description of Additional Supplementary Files

Supplementary Movie S1: The video shows fabrication of semi-cured conductive layer. 95 96

Supplementary Movie S2: The video shows simulation results based COMSOL software for 97 both strain and vibration sensor.

Supplementary Movie S3: The video shows demonstration of ML assisted real-time 100 classification with PSiFI.

Supplementary Movie S4: The video shows digital concierge demonstration using VR 103 environment.
